# An Essay on the Relation of the Theory of Morals to Insanity

**Published:** 1847-07

**Authors:** 


### Art. XVI.

1. *An Essay on the Relation of the Theory of Morals to Insanity.* By T. Mayo, m.d., &c.—*London,* 1847. 8vo, pp. 49.
2. *Elements of the Pathology of the Human Mind.* By Thomas Mayo, m.d., f.r.s.—*London,* 1838. 12mo, pp. 182.
3. *Clinical Facts and Reflections: also Remarks on the impunity of Murder in some Cases of Insanity.* By T. Mayo, m.d., f.r.s.—*London,* 1847. 8vo, pp. 217.

The last of the three works of which we have transcribed the titles, is the only one coming legitimately within the scope of our duty in this Journal; but we have been led to refer to the other two, from the circumstance of our attention being particularly arrested by the chapters in the new volume relating to the Medical Jurisprudence of Insanity. To this last subject we shall exclusively confine our attention at present; hoping to notice the other very miscellaneous contents of Dr. Mayo's last publication, on some future occasion. The greater part of these papers have already appeared in the 'Medical Gazette,' and are therefore not of a novel character; but the members of the profession will thankfully receive them in their collected form, as the productions of an experienced physician, a man of science, and a scholar.

We have long been accustomed to think that a correct appreciation of the relation between criminal responsibility and insanity can only be formed by a more complete analysis of the psychological characteristics of crime and madness, than has yet been effected. No such analysis can be successful, which is not based upon a correct view of the normal operations of the mind, and especially of those which are commonly referred to the *moral* faculties. Both metaphysicians and theologians have been accustomed to speak of the moral sense or conscience as furnishing to every man an invariable rule of right and wrong, any departure from which constitutes a violation of moral or religious duty; and lawyers have taken for granted the existence of such a standard, and have made the test of the sanity or insanity of a criminal to depend upon his supposed ability or inability to distinguish right from wrong at the time of the commission of the offence. Into this doctrine we shall institute as searching an examination as our limits will permit.

The psychical nature of man is usually regarded as composed of intellectual powers, of moral feelings, and of propensities connected (more or less closely) with the animal wants: the first alone constitute the reasoning faculty, which may be exercised independently of the others, as in a mathematical demonstration; whilst the others in all the ordinary affairs of life are concerned in supplying motives to the reason, and are hence the mainsprings of conduct, from which they have received the designation of *active principles.* We have endeavoured on former occasions to show that a further analysis is practicable. All mental operations appear to us to be founded on *simple ideas.* Some of these are purely objects of intellect, exciting by themselves no feelings of pleasure or pain; such are the ideas of time, space, measure, &c. But others are necessarily associated with the simple feelings of pleasure or pain; and thus become (when habitually entertained) the active principles or motive powers, being referrible to the class of moral feelings, or to that of animal propensities, according to the *objects* to which the ideas relate. We cannot, however, see how a clear line of demarcation is to be drawn between these classes. We are accustomed to speak of the *selfish propensities* as differing in their character as well as in their object from the *benevolent feelings:* but we cannot see why amativeness or philoprogenitiveness may not be just as well called a *feeling,* and benevolence a *propensity.* Each is an *habitual idea,* which is dwelt upon and becomes a *motive* to the intellect, because associated with a pleasurable feeling; it is only because the object of the former idea is the gratification of self, and of the latter the happiness of others, that different places are assigned to them in the moral scale. Yet philoprogenitiveness may give rise to actions as disinterested as the purest benevolence; and benevolence may be so indulged at the expense of justice as to become nothing else than a refined selfishness. We consider, then, that such a distinction as the one just alluded to is altogether untenable as the foundation for a system of ethical philosophy; however convenient the terms moral feelings or sentiments, and animal propensities, may be as collective designations.

Now the Motive Powers which are brought to bear upon the Intellect, and which serve to govern the decisions of the Will, are as various as the different classes of objects which excite ideas in the mind; and they are continually liable, therefore, to come into antagonism with each other.

Hence the discussions which every man occasionally holds with himself as to the line of conduct which he shall pursue; his decision being formed according to the preponderance of this or that motive or combination of motives. This is universally admitted to be the nature of the mental operation which we perform, when we are discussing merely the *prudence* of a line of conduct; that is, when we are choosing between two or more lines of conduct into which the question of right and wrong does not enter. Thus we will suppose that we had arranged to take a ride at a certain hour, and the sky in the meantime becomes overcast, threatening us with a wetting if we persevere in our intention; the decision will be governed merely by our estimate of the probability of a shower, our desire to enjoy the anticipated pleasure, and our dislike of the threatened discomfort. But suppose that instead of a pleasure-ride, we are about to set out on a professional visit to a patient whose condition requires our aid; a new motive is introduced, which alters the aspect of the whole question, making our decision no longer one of prudence but of *morality*. Or if, instead of the benefit to another to be derived from our going, we introduce the risk to our own health which may be incurred by exposing ourselves to the rain; we then have another motive which gives the question a moral aspect. And if we suppose *both* these motives to be in action, we may have a very unpleasant conflict between our sense of duty to others and our desire to serve them, and our sense of duty to ourselves and our affection for those who may be immediately dependent upon us. Such a conflict, in regard to exposure to the severity of the weather, infection, or to risk of other kinds, has doubtless passed at some time or other in the mind of almost every one of our readers.

Now it is commonly supposed that, in all such cases, *conscience* or the *moral sense* should be the supreme guide, and that every other motive should be kept in subjection to its dictates. But the difficulty is to determine what conscience directs; and it is surprising to us that, in spite of the continual experience of such a difficulty, both metaphysicians and theologians, to say nothing of the so-called practical men or disciples of common sense, should continue to uphold the *autocratic* nature of this faculty. We have carefully examined the prevalent opinions upon this subject, and find none of them satisfactory. But we have recently met with an analysis of the supposed faculty by an anonymous critic, which perfectly accords with the views we had ourselves previously entertained, and which is so lucidly expressed that we cannot do better than present it nearly in its original form to our readers:*

"If moral good were a quality resident in each action, as whiteness in snow or sweetness in fruits, and if the moral faculty was our appointed instrument for detecting its presence, many consequences would ensue which are at variance with fact. The wide range of differences observable in the ethical judgments of men would not exist; and even if they did, could no more be reduced and modified by discussion than constitutional differences of hearing or of vision. And as the quality of moral good either must or must not exist in every important operation of the will, we should discern its presence or absence separately in each; and even though we never had the conception of more than one insulated action, we should be able to pronounce upon its character. This, however, we have plainly no power to do. Every moral judgment is *relative;* and involves a comparison

* See review of Dr. Whewell's Elements of Morality, in Prospective Review, November, 1845.

of (at least) two terms. When we praise what *has been* done, it is with the co-existent conception of *something else that might have been* done; and when we resolve on a course as *right*, it is to the exclusion of some other that is *wrong*. The fact that every ethical decision is a *preference*, an election of one act as higher than another, appears to us of fundamental importance."

We cannot, therefore, attach a moral character to the actions of animals that are performed under the direction of a blind undesigning instinct, which operates in them as the spring which moves an automaton, leaving them no choice between one course and another; nor can we say that a human action is in itself morally wrong as regards the individual, when it directly results from a violent impulse which he has no power to restrain. But suppose a hungry boy to give his dinner to a famishing suppliant, it is through the operation of pity or benevolence, which makes itself felt in his mind as possessing a higher claim to his attention than his own appetite. His own account of it, in the ordinary phraseology, would probably be that his conscience told him that it would be right to give away his dinner to those who wanted it more than himself. But we agree with the author from whom we have quoted, that, in such a case, pity speaks for itself, when confronted with appetite; and does not want a new power called moral faculty to speak for it. "If, indeed, it acted quite alone, without the presence and competition of any other principle,—if, for the time being, it occupied us wholly, like a solitary impulse, possessing a wild creature,—it would say nothing to us of its worth; but the instant it solicits us with a rival at its side, it reveals to us its relative excellence. And it is the irresistible sense we have, in this case, of its superiority, that is properly denoted by the word *conscience;* the *knowledge with ourselves*, not only of the fact, but of the quality, of our inward springs of action."

According to this view, then, what is termed conscience is nothing else than the *idea of right or wrong* character which becomes attached to an action, when we place in comparison the motives which prompted it; and this idea is entirely dependent upon the relative worth or value in the moral scale, which we have been accustomed to assign to the different classes of motives. The moral rule of action hence consists in the preference of a higher to a lower motive or combination of motives. But it will of course be asked, how are the relative values of these motives to be determined; and the answer is, simply, by the universal consciousness of mankind, which is found to be more and more accordant in this respect, the more faithfully it is interpreted, and the more fully the general mind is expanded and enlightened. It is this tendency towards universal agreement upon this fundamental point, which leads us to feel satisfied that there will in the end be as good a foundation for a science of morals in the psychical constitution of man, as there is for that of music in the pleasure which he derives from certain combinations of sounds. The decisions of the reason, founded upon observation and experience of the results of giving predominance to one or another motive, will aid in the estimation of their relative value; and such aid is most valuable as proving to us the accordance between the different parts of our internal nature and our external position. But it is on our *feeling*, not on our *reason*, that the fundamental axioms of moral science must be based; that feeling being the self-consciousness of the comparative worth of our different springs of action, which guides the reason in the determination of the conduct, not as a distinct autocratic faculty, but as the preponderating result of the comparison which is involved in the very notion of right and wrong.

This is not the place to speak of conscience in its religious aspect; nor is there any necessity for so doing. The introduction of the promises or threatenings held out by Revelation as rewards for good or punishments for evil, simply operates by furnishing an additional set of motives to the determination of our conduct. The desire of the love of God and of the happiness resulting from it, and the fear of His displeasure with its awful consequences, act like any other desires and fears in deciding our will; and their relative force on any occasion will depend upon the degree in which they habitually influence the mind. The complete coincidence between the dictates of the Divine law and the highest principles of pure Morality, prevents one set of motives from ever being placed in antagonism with the other; and the contest generally takes place between these higher tendencies and those lower desires and passions which are in their unrestricted indulgence opposed alike to religion and morality. But there are a great many cases in which it is difficult to decide upon the right course, simply because it is difficult to strike the balance between contending motives; both sides including impulses whose claim to superiority, when acting against those of a low or selfish character, we should at once admit. Thus we have known persons in whose characters the love of *truth* and *justice* are the most prominent features, and who would shrink with horror from any violation of either of these principles for any selfish purpose, sorely perplexed when they have come into apparent collision; the motive to tell or to act a falsehood, being one in itself most just and praiseworthy, namely, the desire to secure the escape of a slave, or in some other way to protect a fellow-creature from oppression or suffering. Now, we do not pretend to decide upon the right course in such a case; we only adduce it in proof of our position, that there is no supreme dominating principle, either in morality or religion, that can invariably and indubitably indicate the rightness or wrongness of a particular action; the moral responsibility attaching to it being entirely determined by the character of the motives which led to the decision. Thus if a man, who might be urged to conceal a fugitive slave near the Canadian frontier, were to refuse to do so merely from the fear of unpleasant consequences to himself, he would be justly branded with the character of a cold-hearted coward; but if his refusal should proceed from the conviction that the divine law requires the preference of rigid truthfulness over every other motive, and that by concealing the suppliant he should be forced into a violation of that law, we cannot blame him, even if we believe that the law of compassion written on our own hearts is at least equally imperative. Similar difficulties beset the upholders of the non-resistance creed, which teaches that *love* is the all-powerful principle in the moral world, and that it should entirely supersede all those impulses of our nature which lead us to oppose force to force, and to resist an unjust and unprovoked assault. Now, here again, we might readily understand and sympathise with those, who consider that the fear of personal suffering does not warrant our doing a severe injury to another in warding off a threatened attack; but when the question comes to be, not of *self*-defence, but of protection to *others* who are helpless dependents on our succour, we feel that the comparative nobility of the latter motive warrants actions which our individual peril might scarcely justify. And in justification of such a course, it might be urged to those who think themselves bound by the Divine injunction "resist not evil," that the context shows that such resistance had reference to individual suffering only, and not to evils affecting others whom the strongest feelings of humanity impel us to guard and protect.

If we are correct in these views, it must be admitted as a general principle, that the ethical judgments of men should be guided, not by any fixed and determined standard of right and wrong applied to *actions*, but by the balance of the *motives* which have led to the actions in question. Under ordinary circumstances it will be easy to strike this balance, when the true motives can be ascertained; from the concurrence of all men, whose minds have been expanded by intellectual and moral cultivation, in their estimate of their relative value. Thus we cannot imagine the circumstances which should induce a woman to sacrifice her chastity, even when the motive is to save the life of one most dear to her. And it is difficult, if not impossible, to put a case in which the violation of truth or justice shall be *clearly* demanded by duty. But notwithstanding the universal horror with which the crime of murder is regarded by all but the most degraded tribes of our race, it is not difficult to conceive the circumstances in which it loses its criminal character and even become justifiable; thus the desire to rid the world of a sanguinary tyrant, at the severest personal risk, has called forth actions which have been in all ages admitted to be noble in their character, even by those who would regard them as prompted by a mistaken principle of duty; and there are few who cannot sympathise with the stern resolve of a Virginius, when paternal affection led him to sacrifice his daughter's life to preserve her honour.

It must be evident, however, that in the construction of laws, the *actions* rather than the *motives* must constitute the foundation of criminal responsibility. It would be impossible for any human power to search the heart in the way that is open to the Divine Being alone; and in by far the greater number of criminal actions, the real motives are very much what they *primâ facie* appear to be. Consequently, in fixing an average of punishment, and leaving it to the administrators of the law to modify or rescind that punishment, in cases in which it can be clearly proved that the motives were of a higher character than the law presumes, enlightened governments adapt their system to the requirements of justice as much as is probably feasible.

Before, however, we proceed to apply these views to the question before us—that of the Criminal Responsibility of Lunatics,—we must offer a few remarks upon the ends of punishment. On this subject a very considerable change of opinion has been gradually taking place in the public mind; and we believe that we are entitled to affirm, that the great majority of those who have duly considered the subject have entirely abandoned the notion that society has any right to inflict punishment as an act of simple retribution—or more properly vengeance—for the offence committed; and are now willing to admit the sole ends of punishment to be the reformation of the offender and the prevention of crime in others.

Some indeed go so far as to assert that these two ends are identical, and that the latter may be disregarded altogether; but while we admit this to a certain extent, in the case of juvenile offenders more especially, we cannot but think that those who uphold the reformation of the offender to be the sole end of punishment, do not adequately estimate the force of the fear of punishment as a motive in determining the conduct of a large class who cannot be influenced and kept in check by higher considerations. Let any one concerned in the education of children attempt to dispense altogether with this motive, and we are very sure that he will find it impracticable to obtain universal obedience, even though he may succeed well with certain natures. Now the great mass of criminals are but "children of a larger growth;" having the higher moral and intellectual nature undeveloped or at least dormant, and acting only with reference to the present gratification of their lower propensities and baser feelings. And there are many more of such, we are well convinced, who are only prevented from becoming criminals by the fear of judicial punishment. It may be said that, where the desire exists, but is thus prevented from exercising itself in action, there is as much moral criminality as if the offence were actually perpetrated; and this must be admitted. But it does not hence follow that the fear of punishment is useless; for if it prevents the commission of meditated crime, society is a great and direct gainer; and it is a much greater indirect gainer, inasmuch as, according to universal experience, the first step in the actual execution of criminal purpose is the almost certain prelude to a downward career of constantly increasing wickedness.

We shall, then, regard the reformation of the offender, and the prevention of crime, as the two great ends of punishment, which are to be made coincident as much as circumstances will admit. And we believe that in the great majority of cases, the latter purpose may be attained without any real interference with the former; a slight modification in the means which experience shows to be most effectual in the reformation of the actual criminal, being frequently sufficient to give the treatment a sufficiently repulsive aspect in the eyes of the would-be criminal, to serve as an efficient motive in deterring him from violation of the law.

Now if it be admitted that there is no fixed and invariable standard of right and wrong, even amongst persons of the highest moral and religious cultivation, who acknowledge the same fundamental principles of ethics and theology, and who recognise the same guides in their application,—and this all experience shows to be the case—it must be further admitted that the standard of right and wrong is subject to much greater variations among those who have never had the higher part of their moral nature properly developed by cultivation, or who have, from original constitution, a want of power to feel the higher class of motives, or who are led by disorder of their intellectual faculties to form a wrong estimate of the respective values of the different impulses which act upon them.

Although the Oliver Twist of Dickens and the Fleur de Marie of Eugène Sue can scarcely be regarded as *impossible* characters, yet it is in the highest degree *improbable* that children brought up in nurseries of vice should remain so free from the most grievous moral pollution as these authors would represent. The moral faculties require no less education than the intellectual; and the early repression of all the higher feelings, and the influences constantly at work to develop the lower, can scarcely do otherwise than fix the standard of right and wrong at a very different point from that which a better education would have determined in the same individual. The young "prig" is led to consider the undiscovered abstraction of a watch as the most creditable action he can perform; and to look at the criminal who "dies game" on the scaffold as his noblest ideal of humanity. "It is absurd," said a superintendent of police, "to talk about the effects of *no* education; for if *you* do not educate these children, the devil will." And if society leaves any portion of the rising generation to this devil's education, what else can be expected than that they should become a pest to the remainder; or how can those beings be regarded as criminally responsible who never knew that they had souls to be saved, or have never experienced such treatment from others, as to have any of the higher moral feelings aroused in their hearts? For such, the duty of the state in our apprehension is most clear; for these offenders against its laws cannot be regarded as subjects of criminal punishment, if they have no consciousness of moral responsibility; and the education which they are receiving, in which the braving of punishment is made to constitute the highest credit, tends to prevent the fear of legal penalties from having any operation in deterring from crime. We look, therefore, to judiciously devised plans for the reformation of juvenile offenders, as amongst the most important means of social amelioration. We believe that the instances will be found to be comparatively few, in which the higher moral feelings are incapable of being called forth by appropriate means, and in which the reason may not be trained into the habitual regard of them as the dominant motives to action. In cases in which, from long repression, or from original peculiarity of mental constitution, these higher feelings are found incapable of being thus developed, and the lower passions and propensities exert themselves without control, we can scarcely regard the individual as a free agent; his condition is more akin to that of brutes; and society has certainly a right to restrain him from the indulgence of his appetites and passions in a way which shall be prejudicial to others, whilst at the same time he has a right to such treatment from society as shall be curative as well as restrictive.

This is the state to which Dr. Mayo has drawn attention in his 'Elements of the Pathology of the Human Mind,' (1838); and it is not inappropriately termed by him *brutality*. He regards it as characterized by the original deficiency or the abolition of the moral sense; and distinguishes it thereby from insanity, in which he considers that there must be intellectual perversion. We cannot agree with him in his definitions; because we do not recognise the moral sense as a single faculty or element, but rather as the collective result of all the higher moral tendencies; and because, moreover, we by no means think that the term insanity ought to be restricted to cases of intellectual perversion. But as to the general features of the condition itself, and the light in which the actions of the individual should be viewed, we are quite in accordance with him. The case of Lord Ferrers affords a striking example of it:

"That nobleman was not *insane* in any customary use of the word; his intellectual faculties were good; and they were directed by a powerful will towards definite objects; neither did he exhibit that moral incoherency which we have described among the earliest phenomena of the insane state. The business-like talents, indeed, which he displayed in his own defence, indisposed his judges to allow him the advantage of *that* plea. But his brutality made him unfit for *social* existence; the laws of this country did not reach him as a subject for confinement. Therefore he was hanged. This procedure was unavoidable under the circumstances of the case, and in the present state of our laws; but it constitutes a painful fact, considering that education at present affords no preventive to such criminality." (p. 132.)

In the Appendix to the same work, Dr. Mayo relates an interesting case, in which he was enabled to carry out his views of the proper treatment of this condition; and we shall quote the description of it, the truthfulness of which will doubtless be recognised by many of our readers from their own knowledge of similar unpleasing specimens of humanity.

"N. B., aged 16, was described to me by his father, who came to consult me in regard to his management, as a boy of singularly unruly and intractable character, selfish, wayward, violent without ground or motive, and liable under the paroxysm of his moodiness to do personal mischief to others; not, however, of a physically bold character. He is of a fair understanding, and exhibits considerable acuteness in sophistical apologies for his wayward conduct. He has made little progress in any kind of study. His fancy is vivid, supplying him profusely with sarcastic imagery. He has been subjected at different times, and equally without effect, to a firmly mild and to a rigid discipline. In the course of these measures, solitary confinement has been tried; but to this he was impassive. It produced no effect.—He was last in a very good seminary in a town in —, where he drew a knife upon one of the officers of the establishment, while admonishing him; and produced a deep feeling of aversion in the minds of his companions, by the undisguised pleasure which he showed at some bloodshed which took place in this town during the disturbances of 18—. He has not appeared to be sensually disposed, and he is careful of property. His bodily health is good, and he has never had any cerebral affection. This boy was, further, described to me as progressively becoming worse in his conduct, and more savagely violent to his relatives. Still I easily discovered that he was unfavorably situated; for his relations appeared to be at once irritable and affectionate; and the total failure of various plans of education was throwing him entirely upon their hands. As an instance of the miserable pleasure which he took in exciting disgust and pain, I was told that, when 13 years old, he stripped himself naked and exposed himself to his sisters." (p. 173.)

It is evident that in this case there was a deficiency of those higher moral feelings, which may usually, by a judicious course of education, be made to keep the lower impulses in check; and among these impulses we think ought to be classed a positive pleasure in giving pain to others, which is the exact opposite of benevolence. This is surely as elementary a tendency as benevolence itself; and is by no means sufficiently accounted for by a deficiency of that disposition and a predominance of destructiveness or other similar propensities. Of the uncontrolled action of this malevolence, in combination with other selfish propensities, we have a most characteristic example in one of the fictitious personages of Mr. Dickens's creation, namely, the diabolical Quilp, the undiluted malevolence of whose nature, perhaps a little overdrawn, manifests itself in almost every action recorded by this veracious biographer; who has not acted with any inconsistency in endowing him with remarkable power of intellect. Such a disposition, of which almost every one may recognise a trace in himself in certain conditions of his system, is peculiarly strengthened by early indulgence; and in a large proportion of the cases in which it is strongly manifested, we believe that it might have been repressed by judicious education. This, however, had not been accomplished in Dr. Mayo's case; and the question had become a very difficult one in what manner the subject of it should be treated. Dr. Mayo recommended that he should be placed under physical restraint; coercion being employed in order to induce him to comply with reasonable requests, and to teach him the desirableness and superior value of self-control; and praise and encouragement being given, as his efforts to acquire it became more successful. Such discipline, as Dr. Mayo justly remarks, should be perfectly free from violence; but should possess that quiet and apparently irresistible force which may give it the passionless character of a law of nature. In the case in question, it was attained by confinement within a private lunatic asylum, the proprietors of which undertook to carry out Dr. Mayo's views; and as the visiting magistrates interposed no obstacle, the experiment was fairly tried, and with remarkable success. It is a curious feature in the conduct of the youth, that he received the magistrates in his apartment without expressing any dissatisfaction with the place, or making the slightest allusion to his *confinement* there: but how far pride contributed to this reserve, or how far the imposing character of the restraint to which he was subjected had impressed him with the idea that to struggle against it would be useless and that patience was his best policy, Dr. Mayo was unable to determine. We should like to know something of the subsequent history of this young man; the immediate result of the discipline was most gratifying, in strengthening his power of restraining himself, and leading him to feel the pleasure of living in an orderly and regulated manner, so that an occasional waywardness was the only remaining symptom of his former condition.

It is much to be desired that establishments could be expressly formed for the reception of such young persons as exhibit the indications of this brutality; whether it arise from early mismanagement or original peculiarity of moral constitution. At present the law requires that they should become qualified by the actual commission of crime for the discipline of a penitentiary; and we have a large number of persons annually arraigned as criminals, who can scarcely be regarded as responsible beings. A great proportion of those who have been remarkable for a long and reckless career of crime seem to belong to this class; but it must be borne carefully in mind, in all attempts to determine criminal responsibility, that the question of departure from that normal or standard condition, in which the higher moral tendencies keep the lower propensities and passions in complete control, is one of *degree* only. We have all of us our besetting sins, of which the world may know little enough, even the closest observer of our external conduct failing to discern their indications; and whatever may be the particular nature of each, it consists in a temporary predominance of some lower motive over a higher, and its permitted exercise upon the will. An action which all regard as criminal results from nothing else; the still lower character of the motive or combination of motives which prompted it, and its more complete opposition to the higher class of feelings, constituting the sole moral difference. On looking over the collection of narratives of remarkable criminal trials, lately translated from the German of Von Feuerbach, we have been forcibly struck with the extreme triviality of the motives which have led to the perpetration of the most terrible crimes; and this not merely singly, but in long succession.

Thus we find Andrew Bichel,—a man not given to the indulgence of violent passions, neither a drunkard nor a gambler, nor quarrelsome, living on excellent terms with his wife, and having a reputation for piety, though at the same time known to be a sneaking pilferer,—killing one young woman after another whom he had decoyed to his house; and for what? Not, as might have been supposed, for the gratification of his lust, that tyrant passion which is the provocative of so many crimes; but merely for the sake of the clothes they wore, which, from their position in life, could be rarely worth more than a few shillings. His master-passion seems to have been avarice; but he was restrained from the indulgence of it by the cowardly disposition which led him to shrink from risk; and it was only when opportunities seemed to present themselves for gratifying it in secret, at the expense of the weak and helpless, that he carried his criminal desires into execution. In describing the murder of one of his victims, he says—"I had a desire to see how she was made inwardly, and for this purpose I took a wedge, which I placed upon her breast-bone, and struck it with a cobbler's hammer, I thus opened her chest, and cut through the fleshy parts of her body with a knife;" and all this, as he subsequently acknowledges, before his victim was actually dead. We can scarcely fancy that a man who could thus commit one murder after another for the sake of a few shillings or pence, which he acknowledges that he did not want, and who could coolly gratify his curiosity about the conformation of the heart and lungs upon the yet living body of the poor girl whom he had just stabbed, after decoying her to his house under the pretence of showing her a magic mirror, could have been endowed with the ordinary feelings of humanity, in a degree that should lead us to regard him as criminally responsible, or at least as a fit subject (so far as he was individually concerned) for the same kind of punishment as that inflicted upon criminals who have received a much higher moral cultivation. The case seems to us to have been one of sheer *brutality;* for though the wretch exhibited a show of piety, it seems merely to have been a thin disguise to hide his real grovelling sordid soul; and his weak will, in the absence of higher motives, must have been completely subjected to the tyranny of a desire which had not in itself strength enough to brave the fear of punishment.

This case, we think a very instructive one; for whilst it leads us to question the justice of the system which inflicts the punishment of death upon offenders who can hardly be considered responsible beings, it also leads us to see the importance, as regards society, of not allowing them to pass scot free on the plea of insanity, and giving to the subsequent measures a character of *mere* salutary restraint. The fear of *punishment* operated as a powerful motive in restraining Bichel from the gratification of his avaricious desires. Had he been a man of more powerful intellect, he might have contrived means for exercising his propensity, without bringing himself within the grasp of the law. But his low cunning merely enabled him to contrive means by which he hoped to elude discovery; and if he had been certain that discovery would have succeeded, his crimes would have never been committed. We fully agree with Dr. Mayo, that "the extent to which the understanding of persons even partially insane may be made instrumental to their self-management, by supplying motives and fears, under dread of punishment, is not at present appreciated." And we believe that the tendency of an enlightened system of criminal legislation, based upon a correct psychology, will be to break down the barrier which at present stands between those who are held excused from all responsibility on the ground of insanity, and those who are deemed fit subjects for the extreme penalty of the law. On the one hand, the treatment of those recognised as criminal lunatics, may be so conducted as to deter from the imitation of their example, instead of suggesting and even encouraging it, as it has frequently done. On the other hand, the treatment of criminals reputed to be sane, will approximate to that which is found most efficacious in the cure of madness. In this manner the courts will be relieved from some of the most trying questions which at present come before them; and the ends of justice will be far more completely satisfied. For example, why should not an insane ward be established in our chief prisons, instead of a criminal ward in our lunatic hospitals? At present, as Dr. Mayo well remarks, in regard to many of the criminal lunatics confined in Bethlem Hospital, "the enduring confinement is in itself a dreadful punishment. But in these cases there is a sad expenditure of unfruitful suffering; for the confinement being entirely and sedulously deprived of the character of a punishment operates unpreventively." And the preventive influence of the restraint is of course altogether lost, when the criminal, being regarded as cured of his insanity, is let loose again upon the world. Dr. Mayo refers to a case noticed in the 'Examiner' for June, 1838; in which the captain of an East Indiaman, who had murdered his crew of eight men in cold blood, but who was excused from capital punishment on the plea of insanity, and placed in the Cork Lunatic Asylum, was subsequently let loose upon the world again. We trust that such may not be the ultimate issue of the trial of Captain Johnson, which most of our readers will remember, who was convicted not long since of the murder of several of his crew, but who was sheltered from punishment by the plea of insanity—a plea chiefly sustained by the extraordinary atrocity of his conduct. Of this individual Dr. Mayo remarks in his visit to Bethlem:—"Captain Johnson's cold, close, subacid visage, and quiet gentle demeanour is awfully contrasted in our recollection with the demoniacal violence which drink and fear, operating upon cruelty, could produce in him, without the smallest warranty of insanity." In the justice of this view of the case we fully coincide. There seems to have been in this man's mental constitution much of the malevolence and vindictiveness which have been already noticed as the chief positive characters of the condition defined by Dr. Mayo as brutality; these were unrestrained by any higher motives; but on the other hand they were fearfully aggravated by habits of intoxication, which not only rendered him most furiously savage whilst under the excitement of alcohol, but doubtless increased the apprehension of personal danger, which he seems to have entertained in the intervals of his temporary madness. We have no doubt that if the fear of punishment could have been duly kept before his eyes, it would have operated as a sufficiently potent motive to self-restraint; but his whole conduct exhibits a coolly-devised scheme to take advantage of the protection which the law most abundantly extends to the captains of ships at sea, for the sake of maintaining the subordination of their crews; and he obviously hoped to be beforehand with his men, by instituting a charge of mutiny against them, which fell to the ground, however, as soon as it became apparent from the concurrent testimony of the passengers and crew, that the captain was the first aggressor. We do not think, as Dr. Mayo seems to do, that this last circumstance "should be viewed as the indication of cunning wickedness, and inconsistent with the supposition of lunacy;" because we believe that in minds decidedly insane, in the ordinary acceptation of the term, very coherent trains of reasoning may be carried out, and a variety of ingenious devices practised, for the sake of gaining a particular end,—especially where that end is the escape from consequences unpleasant to the lunatic. But we do maintain that an enlightened and unfettered consideration of such a case as this, apart from the morbid sympathy for great criminals which has of late been so strangely manifested by the public, would lead to the conclusion that the prisoner was a most fitting subject for the severest punishment which could be inflicted consistently with reformatory discipline; and that this punishment should be of a kind to act as a motive in deterring others from similar conduct. The coincidence between the two cases to which we have last alluded is most striking; and it speaks powerfully of the fearful risk which is involved in the commission of such powers as those which are intrusted to the captains of ships, to individuals in whom the lower class of motives is predominant. The greater the temptation to the abuse of these powers, the more severe and certain should be the penalty for the misemployment of them; and even if an occasional injustice should be done to the individual, by punishing him as if he had possessed entire control over himself, it must be borne in mind that if the discipline, however severe, be curative or reformatory, no permanent injury will be done to him, and society will be the gainer in proportion as the punishment acts as a preventive to crime in others.

Our readers will have seen that we have hitherto avoided all allusion to the question of death-punishment in such cases; but we may now briefly explain our views upon this subject. We do not hold with those who maintain that society has *no right* to take the life of any individual; for if it were demonstrated that capital punishment had more influence than any other infliction in deterring from crimes of certain classes, we should feel justified in upholding it, even though we must thus abandon all idea of reforming the offender. We could be content to leave the fate of the criminal in another world to that Being who searcheth the hearts of men. But from the best evidence we have been able to collect, the punishment of death *has not* this deterring influence; and since the feeling of the intelligent middle classes has been strongly excited against it, so many instances have occurred in which the slightest deficiencies of evidence have been regarded as sufficient grounds for the entire acquittal of the prisoners, or the slightest suspicions or even suggestions of insanity have been held to shield them from responsibility, that the punishment has lost much of its former certainty, and the fear of it necessarily becomes far less operative as a motive for the prevention of crime. We could enumerate many instances of this kind; but they would lead us too far away from our present inquiry. The abolition of death-punishments would be, in our minds, a great step towards that improvement in our criminal legislation to which we look forward;—the treatment of all criminals to a certain extent as lunatics, that is, the devising of a system of discipline in which the reformation of the offender (in other words, his restoration to a wellgoverned state of mind) shall be a no less prominent object than the prevention of crime in others;—and, on the other hand, the treatment of all such as are at present excused from responsibility on the ground of lunacy, as criminals, that is, carrying on the discipline necessary for their restoration to mental health in such a manner as to have the character of a punishment. We are well aware that there are vast difficulties in the way of such a scheme as we propose. Old systems and prejudices have too long interposed barriers to all improvement; and opportunity has scarcely yet been afforded for estimating what may be reasonably anticipated from a change of method,—still less, for determining what are the kinds of punishment which most completely unite the requisites we have indicated. But we are Utopian enough to have an almost unlimited confidence in the efficacy of education,—using that term in its most comprehensive sense,—as regards the prevention of the first crime; and in the efficacy of an improved method of prison discipline as regards the reformation of the offender, and the consequent prevention of future offences by the same individual. It must not be forgotten that the proportion of criminals to offences is really a small one; a very large number of those who have been once committed to prison being recommitted within a twelvemonth after their discharge; and the periods of confinement and liberation henceforth alternating, until some graver offence brings the criminal under the heavier penalties of the law. Even if the reformation of the offender, therefore, could only be accomplished by a system of discipline so mild as not to produce its due restraining influence upon the would-be criminals, we cannot doubt the beneficial effect of such an influence upon society at large. But we see no impossibility in the combination of the two objects; although experience alone can decide in what manner this may be best effected.

Having thus expressed our present views on the much-agitated question of the treatment of criminal lunatics, we may briefly return to some of those more abstract considerations regarding the state of mind that leads to criminal actions, with which we started in the present inquiry; since we wish not to lose the opportunity of recording what appear to us to be the real relations of insanity to crime. As we have already remarked, the emotions, propensities, affections, &c., appear to us all capable of being ranked in the same category; being composed of ideas, with which the simple feelings of pleasure and pain are linked; and being different from each other only in regard to the nature of the objects to which those ideas respectively relate. Any of them, when no longer ranging over the *abstract*, but excited in reference to some *particular* object, constitutes a *desire;* and this desire may be so violent as to constitute a *passion.* The proper province of the desires is to operate upon the intellectual processes, setting these to work to devise the means for their gratification; and the greater part of the actions of our lives are the consequence of motives thus originating in our desires, though they may appear to have been worked out as matters of simple calculation by our reason. But the will is not only concerned in carrying into effect the suggestions of the desires. In the well-regulated mind it ought to have a controlling influence over the desires themselves; so as to prevent them from exercising themselves with undue force. This is, in fact, the power known as self-control; a power which cannot be too early cultivated, or too habitually exercised.

Now we believe that much may be learned by observation of infantile life of the nature of this power. When a young child gives way to a fit of passion, the nurse attempts to restore its equanimity by presenting some new object to its attention, so that the more recent and vivid pleasurable impression may efface the sense of past uneasiness. As the child grows older, the judicious mother teaches it self-control, by calling up in its mind such motives as it is capable of appreciating; the act of self-control being the result of the overpowering influence of the higher motives suggested to it, over the lower or selfish emotions which we desire to bring into subjection. For a time this process needs to be continually repeated; but after a time, a very slight suggestion serves to recall the superior motives to the conflict; and in a further space, under judicious training, they present themselves spontaneously, whenever the evil spirit begins to rise within. We believe it to be a principle recognised by the best educators, that the development of this power of self-control ought to be the object of all nursery discipline; and that appeal should always be made to the *highest* motives which the child is capable of understanding,—punishment being only had recourse to, for the purpose of supplying an additional set of motives where all others fail, and never vindictively inflicted. Now in the exercise of self-control, when once it has been acquired, we believe that the process will always prove to be this:—that the will by a peculiar effort represses the vehemence of one class of motives, by forcibly withdrawing the attention from them, and directing it to another of a higher character. We doubt much the operation of *pure reason* in such a case; since we cannot see how any reasoning can have cogency in repressing a passionate desire, unless it is backed by another motive of the same kind. Thus a man may be deterred from the commission of a crime, by reasoning upon the consequences of the discovery of it with respect to his own worldly position, or to the degradation in which it will involve his family. But he must have a *desire* of the world's approbation, or an *affection* for his family, to give such considerations any weight in his mind. The mind thus swayed hither and thither by various motives contending for the mastery, is at last decided by those which present themselves most forcibly before it; and it is in keeping some in the background and bringing others into clearer view, that the power of the will seems to us to be exerted in modifying the decision,—rather than in simply opposing *reason* to *passion*, which is the ordinary idea of the process of self-control. The reasoning process must always be based upon *motives;* and these motives arise out of the desires, affections, &c., themselves.

But the habit of self-control, unless it is under the guidance of ingenuous impulses, is not unmixedly good. It may be turned to purposes of the deepest dissimulation. Its real operation, however, is the same,—the dominance of one set of motives which the will calls into strength, over another which would have otherwise operated without any restraint to produce an opposite result.

Most crimes result, however, from the undue predominance of some one motive or set of motives (almost invariably those arising out of the selfish desires), and from an insufficient exercise of the regulating power, in keeping these motives in subjection, and in bringing up others of a higher character within view of the mind. From want of education, or from natural inaptitude, there may not be a power of appreciating the force of these higher motives, and the will cannot therefore be expected to decide in their favour. On the other hand we have too many instances of atrocious crimes committed by men who had enjoyed every advantage of education, who had made great moral and religious professions, and who had manifested no deficiency in self-control on all ordinary occasions. The recent murder of Sarah Hart, by John Tawell, is doubtless fresh in the memory of our readers. Now in most of these cases we believe that the *habitual dominance* of the motive which led to the crime may be traced in the previous conduct; and that the will had thus lost much of its controlling power. Thus it is obvious in Tawell's case that desire to stand well with the world by the concealment of his sensual habits, was the immediate cause of the murder of his mistress; and the influence of this desire is obvious in his preceding history, having been the mainspring of many of those actions which the world set down as results of benevolence. Such cases seem to us to border very closely upon insanity; the power of self-control having been gradually lost *quoad* the particular tendencies which at last produced the crime. But even if such a degree of mental disorder were admitted to exist, it could not be held as a sufficient plea in mitigation of punishments, since its *corrective* purpose remains in full force, and its *preventive* object must be equally kept in view, in order that others may not be encouraged in the habitual gratification of their lower desires by the hope of future impunity. So with regard to those paroxysms of anger or of lust, which have led to murders, rapes, &c. in open day and within sight of those who could witness, though they might be too far to prevent, the perpetration of the crime, we believe that they may usually be traced to an *habitual* want of self-control; and that although the individual at the time of committing the crime was so completely under the dominance of passion as scarcely to deserve the name of a responsible agent, he is a proper subject of punishment on account of his *previous* neglect of self-restraint. To treat such a one *merely* as a lunatic, and to absolve him altogether from criminal responsibility, seems to us to be a needless throwing-away of the motives afforded by the dread of punishment to that self-control which *may* be exercised when it is called into action at the right time. Tell a man who is subject to fits of dangerous and really insane passion, that he will be held criminally responsible for whatever injuries he may do to others in his paroxysms, and he will have an additional inducement to endeavour to dispel the storm at its first rising. But let him feel assured that he will only have to lead a gentlemanly quiet life within a lunatic asylum, should he happen to commit murder in one of his outbreaks, and he will have little motive to control himself. The homicidal monomania, as it has been called, passes by such fine gradations into a naturally violent and unrestrained temper, that we cannot see where sanity really ends and insanity begins; and the same may be said of many other mischievous propensities. Thus one of the inmates of the criminal ward at Bethlem is a young woman, who, whilst a nurse in a family at Greenwich, killed the infant which she nursed, and to which she was much attached, under the influence of a wayward and reckless state of mind brought on by a trifling disappointment relating to an article of dress,—just as a fanciful child would break a favorite doll in mere spleen. We have recently heard of a case in which a girl set fire to her master's house no less than six times in one day, without any assignable motive; an act fully as insane as the preceding. And yet to excuse such criminals from punishment, and to subject them merely to restraint, seems to us in complete discordance with the principles upon which the educator and the medical man alike act, when they seek for motives to induce these hysterical subjects to exercise self-control over their wilful and mischievous tendencies.

It is perfectly clear, we think, that in the class of cases on which we have been dwelling, nothing can be more absurd than to set up the offender's knowledge or ignorance of right and wrong, at the time of the commission of the deed, as a test of his criminal responsibility. The clearest knowledge of what is right is not capable of producing abstinence from evil, in individuals in whom the motives supplied by the obligations of moral and religious duty have been habitually neglected, and in whom the baser passions have acquired almost undisturbed sway. On the other hand, individuals in whom the sense of right and wrong has never been sufficiently developed to exert a salutary control over their conduct, may be (in our apprehension) treated in some sort as subjects of criminal responsibility; since the fear of punishment must be held out as a motive to the avoidance of crime on the part of those who are not yet capable of being influenced by any higher motives. But then, let it be remembered, we would have *all* punishments reformatory in its tendency. The impossibility of holding by this standard becomes the more apparent, in proportion to the extent of the survey we take of the different circumstances under which crimes are committed, and of the different moral standards which prevail amongst different communities. Thus the Connaught peasant regards himself as fully justified in ridding himself of a tyrannical landlord by the agency of a musket-bullet; and holds himself bound to shelter and defend any one else who has committed the offence. That the law is against him he knows full well; but *his* principle of patriotic duty leads him to defy the rule of the Saxon, in whatever way it may be exercised; and we do not see how he can be said to do more violence to his sense of right and wrong, than the Briton who deems it right to defend his castle, *vi et armis*, against the midnight robber. Who could feel sure that Balfour of Burley did not conscientiously believe that he was doing God service in the murder of Archbishop Sharpe; or that Charlotte Corday was not animated by so noble a motive, that she has a claim to be regarded as acting up to her notions of right, when she plunged her dagger into the heart of Marat?

Surely all such considerations tend to break down any fancied barrier that the ingenuity of man can devise between sanity and lunacy, so far as relates to criminal responsibility. No general test can be applicable to all cases. In the great majority of the crimes committed at the present day, it is true, the question of insanity is not raised, and needs not to be, if the views we have expressed of the objects of punishment are carried out, as the present administration seems endeavouring to do. Yet if the *vindictive* system of punishment remained in force, we should unhesitatingly raise the plea of insanity in regard to by far the greater proportion of offenders brought to trial in this country; on the ground that their education has been such as to draw forth their worst passions instead of their purer feelings,—that their sense of right and wrong is formed upon a standard so opposite to ours that they had much better have none at all,—and that their intellect, having been exercised only in the gratification of the most vicious desires, is not capable of being directed in any other channel, until an entirely new set of motives is aroused for its exercise. How could a child born in the garrets of St. Giles's, the offspring of a prostitute and a thief, who is brought up to his father's profession, and follows it with a dexterity which he regards as highly creditable, be placed in the same category with the son of moral and religious parents, who has been educated in the midst of domestic happiness, has been led to feel every conceivable motive for perseverance in the path of rectitude, and has all his reasonable wants gratified,—but who steals or forges to gratify some low sensual taste, which has grown into a dominant motive from the licence accorded to it? Surely if society feels it right to execute *vengeance* on the criminal, the *latter* is the one on whom it should fall; for what right has society to *punish* for dereliction of duty the criminal who has never received at its hands the opportunity of acquiring any higher standard than that to which he believes himself to have acted up?

Again repeating that all the difficulties of the subject appear to us to be removed by a due appreciation of the ends of punishment and a proper adjustment of it to the two great purposes of the prevention of crime and the reformation of the offender, we leave the subject for the present in the hands of our readers.

---

